# Ethics and bias in emotional AI

**DOI:** 10.3389/frai.2026.1768696

**Published:** 2026-03-05

**Authors:** Smrithy G. S, Balaji Chandrasekaran, Omana J

**Affiliations:** 1 School of Computer Science and Engineering, Vellore Institute of Technology, Chennai, India; 2 Department of Computer Science and Engineering, Anna University, Chennai, India

**Keywords:** AI governance, algorithmic fairness, emotion recognition, privacy, transparency

## Abstract

Emotional Artificial Intelligence (Emotional AI) is a branch of artificial intelligence that combines machine learning, natural language processing, and computer vision to perceive and react to human feelings. Emotional AI will enhance more intuitive and personal human-machine interactions by analyzing facial expressions, speech patterns, physiological factors, and behavioural expressions, and find applications in healthcare, education, customer service, and other fields. Although this field is promising, it comes with serious ethical issues especially on privacy, transparency, accountability and fairness. The nature of human emotions is intricate, context-specific and culturally biassed, thus the perceptions of emotions are challenging and subject to biasness in the perception. Besides, emotional data is sensitive, thus, causing concerns over its abuse, surveillance, and infringement of individual rights. The problems of algorithms bias, the representativeness of data, and even fairness also make the implementation of the Emotional AI more problematic since biassed systems can serve to strengthen stereotypes and inequalities in society. This perspective explores the ethical issues of Emotional AI, which brings out the need to develop ethically, establish good governance, and work together internationally to ensure that Emotional AI is used in a way that benefits humanity without denting human dignity, security, or social justice.

## 1 Introduction

Emotional artificial intelligence, often known as emotional computing, is the subset of artificial intelligence enabling machines to recognise, evaluate, and respond to human emotions. To evaluate emotional indications acquired from facial expressions, voice tone, physiological data, and behavioural patterns, this technology combines many computing technologies including machine learning, natural language processing, and computer vision. Unlike traditional artificial intelligence systems that give logical problem-solving and data-driven decision-making first priority, Emotional AI aims to link human emotions with technology thereby improving interactions with computers to be more intuitive and customised to the psychological state of the user. To enable more human-like digital interactions, developers want to include emotional intelligence into artificial intelligence systems, thereby boosting user experience, communication, and personalised offers across many spheres (Jhamb and Ryan, 2022).

Emotional artificial intelligence is essentially about the ability to understand and evaluate nonverbal signs people use naturally to express emotions (Domnich and Anbarjafari, 2021). Micro-expressions and subtle muscle movements identified by facial expression recognition technologies allow AI systems to discern an individual’s emotional state—that of pleasure, sorrow, rage, or surprise. Emotional artificial intelligence depends critically on voice analysis as speech patterns, tonal variations, pitch, and rhythm frequently convey emotions that may not be explicitly expressed by words. Particularly in applications related to stress management and healthcare, physiological signals—such as heart rate variability, skin conductance, and eye movement—offer more understanding of an individual’s emotional state. Emotional artificial intelligence increases the accuracy of emotion identification and supports real-time assessments of human emotions by combining these many senses.

Emotional artificial intelligence mostly seeks to create more sympathetic technologies that can understand human emotions and respond appropriately. AI-driven chatbots and virtual assistants with emotional intelligence can identify frustration or discontent in a user’s voice and change their responses in customer service applications, either by offering help in a more comforting manner or escalating the matter to a human representative. Emotional artificial intelligence is used in education to generate tailored learning experiences by means of facial expression and engagement levels, therefore allowing teachers to modify their techniques depending on real-time emotional input. Emotional artificial intelligence is having a significant impact on the healthcare industry, particularly with regard to mental health monitoring and treatment. AI-driven systems can assess emotional pain via speech and facial signals, therefore enabling early detection of anxiety or depression and quick fixes.

Emotional artificial intelligence still is an emerging field full with ethical conundrums and various issues throughout its development. Given that human emotions are complicated, contextually sensitive, and moulded by cultural and personal differences, robots have limited ability to correctly sense emotions. The privacy and security of emotional data is a topic of constant debate as artificial intelligence systems need the processing of rather sensitive personal data to function as intended. For researchers and developers, ensuring that emotional artificial intelligence operates ethically—that it does not reinforce stereotypes or be exploited for manipulative purposes—is a great difficulty.

Although these ethical issues are relevant to artificial intelligence in general, they have an increased impact in Emotional AI, because of the close and interpretive character of emotional information. The closer the artificial intelligence is to various aspects of everyday existence, decision-making, and other critical sectors, the greater the significance of ethics in the field of AI technology. Artificial intelligence (AI) can process high volumes of information, streamline multi-faceted tasks and deliver insights that influence policy decisions, business strategies and individual decisions. Nevertheless, this enormous strength demands ensuring that AI works fairly, transparently, and without injury. Ethical issues in artificial intelligence are needed to reduce prejudices, protect privacy, guarantee responsibility, and preserve human dignity. Artificial intelligence can support discrimination, infringe human rights, and cause unforeseen consequences that will undermine the entire society without any ethical limitations.

Justice and bias are the two important ethical concerns in artificial intelligence. AI systems are trained based on previous data, so once the training data is biassed, the AI will reproduce it and potentially aggravate the preconceptions. The recruiting systems, the credit scoring systems, and the facial recognition technology have been discriminating against gender, race and socioeconomic status. To achieve fairness in artificial intelligence, it is required that large, representative datasets are used, that it is extensively tested to identify biases and that it is continuously monitored to prevent biassed results. Rather than reinforcing the current biases, developers should fiercely develop AI systems to reinforce equality and diversity.

The privacy problem is a major ethical concern in the field of artificial intelligence technology since most of the AI-driven applications depend on the collection and analysis of large amounts of personal data. In use in smart homes, social media, banking, or healthcare, artificial intelligence (AI) can be used to access sensitive data and thus casts doubt on the issue of data security and consent. Misuse or mismanagement of personal data may result in breaches, unlawful surveillance and intrusion into the privacy of person. In order to make sure that artificial intelligence systems do not breach personal freedoms and rights, ethical AI creation presupposes the stringent regulations on data protection, transparent user authorisation processes, and adherence to privacy laws.

Openness and responsibility is a key ethical concern in artificial intelligence. A large number of artificial intelligence algorithms are black boxes, i.e., their decision-making process is not easily comprehensible and opaque. The absence of openness may be adverse, particularly in key sectors like financial services, healthcare and criminal justice where AI is applied. The victims of artificial intelligence decisions must be able to learn about the logic and method of such decisions. Transparency requires the creation of Explainable Artificial Intelligence (XAI), where in that situation human supervision has been incorporated into AI-powered systems and algorithms have rational explanations of their outputs. In addition, there must be a definition of responsibility so that in case the AI systems fail or harm people, there is a well-defined procedure to define responsibility and take corrective actions.

The impact of artificial intelligence on employment and the labour market adds even another ethical question. AI raises questions about job displacement and the future of work even if it may improve efficiency and automate boring tasks. Businesses have to strike balance as artificial intelligence develops between protecting workers from obsolescence and employing technology to improve output. Adoption of ethical artificial intelligence calls for policies stressing human-AI cooperation over complete job displacement, workforce transition initiatives, and attempts at reskilling.

Apart from personal users, ethical consequences of artificial intelligence include worldwide concerns like AI’s participation in war, spread of misinformation, and autonomous decision-making. Using artificial intelligence in military settings, for example, raises serious ethical questions concerning autonomous weapons and assigning life-or-death decisions to robots. Likewise, public trust and democracy are seriously threatened by AI-generated deception including automated news production and deepfake technologies and automated fabrication of objects. Good ethical AI governance calls for worldwide cooperation, strict policies, and ethical frameworks stressing human safety, accuracy, and responsibility.

This Perspective argues that Emotional AI systems rest on a fragile assumption: that human emotions can be treated as stable, measurable ground truth signals suitable for automated inference and decision-making. We contend that this assumption introduces systematic ethical risks across the AI pipeline—from data labelling and model training to deployment and governance. By synthesizing documented deployments and governance literature, this article proposes a structured lens to understand bias pathways, measurement uncertainty, and accountability gaps unique to emotion inference technologies.

## 2 Scope and approach of this perspective

This article adopts a conceptual and case-anchored perspective approach. It does not report new empirical experiments, nor does it conduct a systematic review. Instead, it critically examines documented Emotional AI deployments and representative governance literature to highlight recurring ethical risks, bias mechanisms, and measurement limitations.

## 3 Ethics in emotional AI

Due to the growing nature of artificial intelligence in machines that assess, identify, and react to human emotions, Emotional AI is a significant topic in terms of ethics. Emotional artificial intelligence offers both a lot of possibilities and significant ethical dilemmas using facial expression recognition, voice analysis and physiological data to determine human feelings. Real-time emotional recognition of AI may lead to improved user experience in education, marketing, customer support, and healthcare and in manufacturing. Nevertheless, the ethical implications of transparency, data protection, and privacy must be thought through to ensure that no one misuses the transparency, discriminates against and affect others in an unexpected way. The introduction of bias in Emotional AI can occur upstream prior to the release of emotion inference into decision-making systems via data collection, labelling practices and model design choices.

The artificial intelligence of emotions poses a significant ethical issue in terms of privacy and data security. Emotional artificial intelligence systems may often demand very sensitive personal information such as facial images, voice recording, heart rate data, and behavioural pattern. The inappropriate information collection and storage is a danger to individual privacy. Unauthorised access to this data or breach of data or even lawful but unethical use of the data may have significant implications such as bias, surveillance, and manipulation. Targeted advertising by companies using Emotional AI may obtain and analyse the emotions of people without necessarily knowing it, therefore, creating intrusive and exploitative marketing techniques. Furthermore, the use of emotional artificial intelligence by companies to monitor the emotions of their employees in the business world may pose an ever-surveillance scenario, thus violating human freedom and privacy. Ethical emotional artificial intelligence research should put high priority on data security methods such as anonymization, encryption, and stringent access controls to assure that the personal emotional information of people is not violated. Additionally, AI-related companies and developers must establish formal guidelines on user authorization, which will guarantee that consumers are sufficiently versed about the collection, processing, and storage of emotional information (Deotale et al., 2025).

Emotional artificial intelligence raises serious ethical questions about transparency in AI decision-making processes as many AI systems function as “black boxes,” meaning that users or even designers cannot easily understand their decision-making mechanisms. When Emotional AI is used in vital industries such healthcare, employment, law enforcement, and education, the lack of openness raises significant ethical concerns (La Porta et al., 2025). For example, it is important to understand how the AI system views such emotions and whether its assessments are based on scientifically supported criteria if it evaluates a candidate’s job fit based on their emotional reactions during an interview. AI-driven emotional assessments used in mental health apps have to be transparent so that users may understand the analysis of their emotional condition and find the dependability and objectivity of the AI’s decisions.

Transparency in emotional artificial intelligence calls for the development of explainable AI models with unambiguous, understandable reasoning for their judgements. Users should be informed not just on the data sources utilised by artificial intelligence but also on the approaches used by the system to analyse and interpret that data for emotional assessments. Prevention of biases resulting from ethnic, gender, or cultural variations in emotional expression depends on openness. Many artificial intelligence models are created utilising datasets that could lack complete representation of diverse populations, therefore producing biassed interpretations. Training biases in predominantly Western datasets have revealed facial recognition systems to mistakenly detect emotions in people from many ethnic backgrounds. Should artificial intelligence exhibited systematic demographic accuracy disparities resulting from inaccurate or inadequate data, it might lead to discriminatory effects, hence aggravating the marginalisation of certain groups (Aalam et al., 2025).

To tackle these ethical difficulties, companies developing Emotional AI must set ethical AI governance structures that give justice, responsibility, and transparency top priority. Ethical standards must demand that consumers have open knowledge about how AI systems work and have the ability to challenge emotional assessments produced by AI when called for. Furthermore, regulatory regulation is necessary to ensure that companies using Emotional AI follow ethical standards and avoid leveraging customer emotions for political, financial, or surveillance purposes. Recent regulatory initiatives, such as the European Union’s AI Act, explicitly recognize emotion inference systems as high-risk applications, emphasizing the need for transparency, human oversight, and bias mitigation (European Parliament and Council of the European Union, 2024).

## 4 Bias in emotional AI

Another significant issue in Emotional AI is the bias in artificial intelligence that is supposed to assess human feelings on the concept and has some impact on the ethical implications, accuracy, and equality. Emotional artificial intelligence involves the artificial intelligence method of applying machine learning to interpret facial expressions, vocal tones and physiological cues to understand the emotion of an individual. Nevertheless, such algorithms are likely to become affected with numerous biases that might lead to unfair or erroneous results. Data collection, algorithm design and subjective nature of human emotions might impose constraints on emotional artificial intelligence and cause bias. Poorly regulated biassed emotional artificial intelligence systems could support the pre-existing socioeconomic disparities and cause biassed decision-making processes in areas such as employment, medical care, law enforcement, and customer service.

The primary form of bias that is observed in artificial intelligence systems is dataset bias. The quality of the training data used by the AI system relies on the quality of materials upon which the information is built; hence, when the datasets lack healthy representation of the varied populations, the models developed could present biassed results. A lot of the facial expression recognition systems are trained on the data that primarily contains people who belong to a particular racial or ethnic groups. In case the dataset significantly represents the western facial expression, the AI model may fail to constantly interpret the emotions of non-western individuals. This makes mistakes in emotional recognition that the algorithm might falsely recognize emotion, or overlook subtle expressions that are culturally specific. Gender prejudice in emotional AI is very high because it has been noted that many artificial intelligence algorithms interpret emotions differently based on gender stereotypes. As an example, even though the manifestation of emotions of men is considered to be more neutral or even angry, whether or not the communication of emotional state is accurate, the facial expression of women is more likely to be considered as being happy or emotional. These attitudes may support preconceptions and cause unfair treatment in such areas as a psychiatric assessment procedure or employment.

Another key form of bias in artificial intelligence of emotions is algorithmic bias where the mathematical models of artificial intelligence decision making are designed to prefer certain patterns to others or the mathematical models themselves are erroneous. The algorithm can give biases towards some phrases or even voice tones despite the diversity in a dataset hence creating systematic errors. Most artificial intelligence models, such as those which predict or guess at emotions using exaggerated gestures, assume that a big grin necessarily means one is happy or a scowled face necessarily that one is angry. Nevertheless, human feelings are so complex and individuals do not always express them. Whereas certain cultures emphasize the outward displays in social contexts, certain ones endorse the hiding of intense feelings. A machine-learned design that is biassed towards an expression of a particular type of emotion may be confused by those who do not conform to that norm. This could have significant implications in areas such as mental health testing, where AI is becoming more popular in the detection of depression or anxiety. A biassed artificial intelligence system can ignore evidence of suffering in individuals whose emotional responses do not fit into the pre-determined categories of emotion, thus resulting in wrong diagnosis or insufficient support.

Emotional artificial intelligence cannot withstand interpretation bias, which is inherent to human emotions since they are complex and situational. The artificial intelligence systems often tend to assess the feelings independently without paying attention to the overall history of the human life, his/her upbringing or experience. The absence of the contextual comprehension may cause misguided impressions. Even though the person can present facial expressions that artificial intelligence can read as a sign of anxiety or hopelessness, it can be possible that he or she is simply tired or deep into his or her thoughts. The emotional expressiveness may be misinterpreted due to the differences in the cultural backgrounds. In some cultures direct eye contact can be interpreted as confidence and honesty and in others, it can be interpreted as scorn or hostility. An artificial intelligence system trained to perceive emotions according to one culture criterion can be used to analyze individuals that have different backgrounds, giving unfair or untrue observations.

Reducing bias in Emotional AI calls for an all-encompassing approach including improved dataset diversity, algorithmic architectural optimisation, and integration of contextual awareness into AI systems. Training sets must be guaranteed to comprise a wide range of demographic groups, thereby allowing for variations in age, gender, ethnicity, and cultural background. Before they are put into use, artificial intelligence systems have to go through bias testing using approaches such adversarial testing and fairness audits to find and correct any disparities. Furthermore, Emotional AI models have to be designed to provide logical explanations for their findings so that users may understand the evaluation of emotions and have opportunity to challenge or correct misunderstandings (Wang et al., 2020). Guaranturing that artificial intelligence systems do not operate autonomously without monitoring and responsibility depends on human oversight.

## 5 Case studies illustrating ethical risks in emotional AI

This section presents selected real-world case studies to illustrate ethical risks associated with the deployment of Emotional Artificial Intelligence and related automated decision systems. The cases are not intended as exhaustive empirical evaluations but as documented examples highlighting recurring issues of transparency, bias, privacy, and governance. Each case is analyzed using a consistent ethical lens to clarify decision impact and lessons for responsible Emotional AI design.

### 5.1 Case study 1: HireVue and emotion-based hiring

HireVue is an AI-based recruitment platform that employed emotion inference techniques—including facial expression analysis, vocal characteristics, and linguistic cues—from recorded video interviews to support hiring decisions. Renowned AI-based recruitment tool HireVue came under fire in 2019 for using emotional artificial intelligence to assess job prospects. Based on responses in recorded video interviews, the company evaluated prospective hopefuls using facial expression recognition and voice analysis (Harwell, 2019). Designed to assess candidates’ work fit by assessing their voice tone, facial expressions, and word choice, the artificial intelligence system assigns scores that would influence employment choices. Though it raised important ethical issues about privacy, justice, and transparency, the technology meant to maximise the hiring process and reduce human bias begged serious moral considerations.

In the given instance, transparency in the decision-making process of AI system was the significant ethical issue. There was also the problem of candidates making it difficult to challenge or appeal the results where they were unaware of the specific criteria applied to rate their facial expressions and spoken patterns. Notably, there were no direct explanations given to the applicants on how particular emotional cues were prioritized or how end suitability scores were created, which constrained their opportunity to challenge or put into context automated assessments. The technology was a black box, even HireVue staff failed to explain the method used in giving grades in recruitment. Absence of transparency made candidates who had unjust evaluations without the instruments to interpret or transform their scores. Moreover, the technology threatened to make other stereotypes instead of breaking them. Facial expression recognition systems have been found to be difficult when it comes to relatively measuring emotions cast among several ethnic, gender, and cultural groups. According to the studies, artificial intelligence algorithms are prone to misunderstanding the emotions of people with a darker colour, and hence, may endanger the minority candidates.

One major issue was invasions of privacy. Candidates were required to provide video recordings so that the AI could compile and evaluate their facial expressions and vocalisations free from complete understanding of their usage or storage of this data. This caused concerns about data security, especially in view of the growing danger presented by artificial intelligence-driven surveillance and possible biometric data misuse. Critics said that companies may be using emotional artificial intelligence for customer profiling or worker monitoring without permission, therefore transcending recruiting.

The application of emotional artificial intelligence by HireVue has attracted criticism that has led to a public debate and legal analysis. Ethicists and privacy campaigners of artificial intelligence cautioned that job evaluations largely based on AI could discriminate against those whose emotions display differently based on neurological conditions, disabilities, or cultural differences. To overcome the continuous criticism and regulatory pressure, the HireVue company published a statement in 2021, stating that they would no longer use facial analysis in its hiring tests, instead switching to evaluation of verbal and written answers.

The HireVue case emphasises the moral dangers of emotional artificial intelligence, particularly in important settings like hiring. More transparency, more strict laws, and better AI model design will help to solve these moral conundrums. Companies utilising emotional artificial intelligence have to make sure candidates and users are adequately informed on the data collecting and processing process. To find and reduce prejudices in their decision-making processes, artificial intelligence systems must go through fairness audits. Moreover, authorities of regulations have to provide clear guidelines for the moral application of emotional artificial intelligence so as to ensure that it does not lead to discrimination, invasions of privacy, or dubious decisions. This case demonstrates how opaque emotion inference in high-stakes contexts such as recruitment can undermine fairness and accountability, particularly when emotional assessments are treated as objective indicators of suitability.

### 5.2 Case study 2: demographic bias in facial analysis systems

Although the Gender Shades study primarily examined demographic disparities in gender classification rather than direct emotion recognition, its findings are highly relevant to Emotional AI. Emotion recognition systems rely on the same facial feature extraction pipelines, datasets, and labelling practices, meaning that demographic accuracy gaps in facial analysis directly translate into ethical risks for emotion inference technologies.

Researchers at MIT and the University of Toronto identified significant racial and gender disparities in commercial facial analysis systems, particularly in gender classification accuracy across demographic groups (Buolamwini and Gebru, 2018). The study examined widely deployed facial analysis tools developed by major technology providers and demonstrated systematic performance gaps based on skin tone and gender. While the study did not evaluate emotion recognition directly, its findings are highly relevant to Emotional AI because facial analysis pipelines constitute a foundational input layer for many emotion inference systems. When such facial analysis systems are incorporated as upstream components in emotion recognition pipelines, these demographic accuracy disparities propagate downstream, increasing the risk of biassed emotion inference in high-stakes applications such as hiring, surveillance, and behavioural assessment.

The central discovery of the study was that facial analysis systems tended to err in the classification of the expressions of the black people, more often giving the participants the high marks in such emotions as fury and aggression, but with a neutral facial expression. In contrast, the white people could be more commonly characterized as showing pleasant emotions even in questionable circumstances. This tendency caused major ethical and practical issues, especially in the context of the application of the emotional artificial intelligence to recruiting, security surveillance, and law enforcement. In case an artificial intelligence-based recruitment model identifies the black applicants as more aggressive or more untrustworthy in their emotional recognition, which is biassed, it may end up penalizing them unfairly in job opportunities. Unbalanced AI-enhanced surveillance in the criminal justice system may lead to the focus on minority individuals, thus, endorsing institutional bias and racial profiling. Misclassification risks are magnified when these biassed facial analysis pipes are introduced into emotion recognition models, especially when it comes to scenarios of surveillance, hiring or behavioural evaluation.

These prejudices started from the datasets used to train the artificial intelligence programmes fundamentally. Many emotional artificial intelligence systems are built using datasets largely including white people, producing models that learn and improve their recognition patterns depending on a limited range of facial expressions and emotional signals. Therefore, when these algorithms assess faces that depart from the main dataset, they might provide false conclusions. Furthermore, the models neglected to appropriately address cultural differences in emotional display, therefore aggravating the problem. Different cultures have different ways of expressing emotions; hence, a system taught mostly on Western facial expressions may find it difficult to consistently recognise emotions in folks from non-Western backgrounds.

Many approaches have been proposed and tried to help to overcome these difficulties. A first approach is to improve the representativeness and diversity of training sets. To reduce bias, artificial intelligence researchers and developers have to ensure that their models are trained on a wide spectrum of faces from many racial, gender, and cultural backgrounds. Since then, companies like Affectiva have worked to extend their databases to include people from different backgrounds, realising that a more inclusive dataset produces better and more fair AI performance.

Another option is conducting bias tests and fairness audits before emotional artificial intelligence systems are put to use in useful applications. Independent scholars and regulatory bodies have to evaluate artificial intelligence models for bias and identify any differences in accuracy across several demographic categories. Developers have to improve their algorithms to ensure fair outcomes after finding prejudices. Furthermore required is more openness in AI decision-making procedures. Users and stakeholders have to be informed about the approaches used in artificial intelligence-driven emotional analysis, the datasets utilised, and the natural technological limitations.

Reducing bias in emotional artificial intelligence depends critically on ethical standards and regulatory systems. Governments and international agencies have started suggesting structures to ensure that artificial intelligence systems follow standards of responsibility and fairness. Especially in high-risk fields like recruiting and law enforcement, the European Union’s AI Act emphasises the importance of reducing bias in AI-based decision-making. Companies developing emotional artificial intelligence technology have to follow guidelines to ensure their models are moral and exact. This case highlights that bias in Emotional AI often originates upstream, at the level of data representation and facial analysis accuracy, rather than solely within emotion classification models themselves.

### 5.3 Case study 3: Amazon’s automated hiring system (adjacent case)

While Amazon’s automated hiring system did not perform emotion recognition, it is included here as an adjacent case illustrating how biassed data and automated decision pipelines can produce discriminatory outcomes—risks that would be exacerbated if emotional inference were integrated into similar systems.

When Amazon revealed in 2018 that it had dropped an internal artificial intelligence system meant to support hiring, the company faced a lot of criticism (Dastin, 2018). Designed over several years, the system sought to maximise Amazon’s hiring process by automating resume reviews and providing analysis on candidate fit. It was revealed that, particularly for technical roles, the artificial intelligence system showed natural discrimination against female candidates. This prejudice started with the training data used to build the algorithm, which largely consisted of resumes from men, therefore mirroring the historical male dominance in Amazon’s technology team. The algorithm thus preferred resumes that closely matched those of its mostly male workforce, punishing resumes including terminology usually associated with female candidates, such “women’s” or “female,” while undervaluing experience in roles traditionally occupied by women.

The unintended result was that, despite women having same qualifications and experience, the algorithm ranked resumes from women lower than those of men. Apart from gender prejudice, the system showed a pred inclination for several language patterns, particularly more aggressive and competitive language, which matched the dominant male preconceptions in the computer industry. Given the increasing dependence on artificial intelligence in important decisions like hiring, Amazon’s use of it for automated employment decisions creates a disturbing situation wherein the company may reject gifted female candidates due to biassed data, therefore posing serious questions.

When Amazon’s HR team discovered the discrimination, they stopped the system and acknowledged the issue, therefore confirming that the model was never designed for production deployment and that they had neglected the issues given by the bias. The company claimed that the AI’s decision-making depended on past employment patterns that preferred male candidates, therefore promoting systematic discrimination. Still, Amazon did not completely discount artificial intelligence throughout the hiring process. The company appreciated the knowledge acquired from the event and started to create better and more inclusive AI models competent of being trained on a large spectrum of data.

Improving data variety was Amazon’s main cure after the event. Amazon started the curation of more inclusive training datasets guaranteeing a balanced representation of both male and female candidates across several technical fields in order to reduce the bias resulting from male-dominated historical data. They used information from many different industries and job roles to ensure the artificial intelligence was not just mirroring past hiring patterns but could also assess a wider range of applicant traits. By switching to more representative data, Amazon was able to create artificial intelligence models showing reduced sensitivity to gender prejudice.

Amazon has took steps to add human control right through the hiring process. The company applied employing a hybrid approach, incorporating human recruiters in latter stages of the hiring process and artificial intelligence for early resume screening. By means of human judgement, this hybrid approach helped early identification and correction of biases in the artificial intelligence, thereby preventing unfair decisions resulting from erroneous data. Amazon also started regular audits of its artificial intelligence systems to ensure any potential biases could be found and corrected before the technologies were put to use in useful applications.

The case of Amazon and its artificial intelligence system show the danger of overreliance on machine learning algorithms without considering more broad ethical concerns. Among the critical things that were learnt was the fact that artificial intelligence systems are reliant on the quality of the data one employs in its development. The danger of the artificial intelligence systems reinforcing the existing injustices is massive by overlooking the previous biases and disregarding other arguments. This episode helped to understand why transparency and responsibility are essential in making decisions based on artificial intelligence when companies were forced to acknowledge errors and take corrective measures when the presence of prejudice was revealed. The case of Amazon highlights pertinence of human control, representativeness of data and stable auditing as the principles of governance that are equally relevant to the ethical implementation of Emotional AI systems.

Collectively, these cases demonstrate that ethical risks in Emotional AI and related automated systems are not isolated technical failures but structural consequences of data bias, opacity, and questionable assumptions about human behaviour. These challenges are further intensified by the difficulty of defining and validating emotional ground truth, which raises deeper questions about the measurement and interpretation of emotions in AI systems.

## 6 The measurement problem in emotional AI

Emotional AI systems rely on the assumption that emotions can be objectively labelled and inferred from observable signals such as facial expressions, speech, or physiological responses (Barrett et al., 2019). However, emotions are context-dependent, culturally mediated, and often ambiguous, raising fundamental questions about the validity of emotional ground truth. Treating inferred emotions as stable and measurable entities risks reifying uncertain labels into authoritative decision inputs (McStay, 2018). This measurement problem has direct ethical implications for fairness, accountability, and transparency, particularly when Emotional AI is deployed in high-stakes domains such as hiring, healthcare, or surveillance.

## 7 Conclusion

The potential of emotional AI lies in the possibility that it will transform the nature of human-machine interaction by allowing systems to process and react to emotions in more compassionate, customised as well as context-sensitive manners. Nevertheless, the very powers that have enabled Emotional AI are also very dangerous in terms of morality. The gathering and processing of emotional information put users at risk of privacy invasion, surveillance, and manipulation, and the biases in datasets have the potential to continue discriminating against them in values like recruitment, education, and healthcare. The process of developing Emotional AI ethically must be thus guided by a conscious emphasis on equity, openness, responsibility and human interests. The developers and policymakers need to introduce protective measures including explainable AI, strong privacy controls, and international regulations to practice responsible innovation (UNESCO, 2021). In the end, the difficulty does not just consist in the rise of technological capacity, but also in the incorporation of ethical values safeguarding individual rights and fostering fairness. When approached with care, Emotional AI may become an empowerment instrument and a means of greater well-being; otherwise, it will become a threat to further social stratification and the lack of trust in technologies.

## Data Availability

The original contributions presented in the study are included in the article/supplementary material, further inquiries can be directed to the corresponding author.
